# Ferroferric Oxide Significantly Affected Production of Soluble Microbial Products and Extracellular Polymeric Substances in Anaerobic Methanogenesis Reactors

**DOI:** 10.3389/fmicb.2018.02376

**Published:** 2018-10-09

**Authors:** Qidong Yin, Kai He, Shinya Echigo, Guangxue Wu, Xinmin Zhan, Hongying Hu

**Affiliations:** ^1^Shenzhen Environmental Science and New Energy Technology Engineering Laboratory, Tsinghua-Berkeley Shenzhen Institute, Shenzhen, China; ^2^Key Laboratory of Microorganism Application and Risk Control of Shenzhen, Graduate School at Shenzhen, Tsinghua University, Shenzhen, China; ^3^Research Center for Environmental Quality Management, Kyoto University, Kyoto, Japan; ^4^Department of Environmental Health, National Institute of Public Health, Wako, Japan

**Keywords:** DIET, conductive materials, soluble microbial products, extracellular polymeric substances, electron shuttles

## Abstract

Conductive materials facilitate direct interspecies electron transfer between acidogens and methanogens during methane (CH_4_) production. Soluble microbial products (SMP) and extracellular polymeric substances (EPS) produced by microorganisms might act as the electron shuttle between microorganisms and conductive materials. In this study, effects of conductive ferroferric oxide (Fe_3_O_4_) on anaerobic treatment process and the production of SMP and EPS were investigated. The maximum CH_4_ production rate was enhanced by 23.3% with the dosage of Fe_3_O_4_. The concentrations of proteins, polysaccharides, and humic substances in tightly bound EPS (T-EPS) were promoted, suggesting that extracellular metabolisms were induced by conductive materials. Distribution of potential electron shuttles such as quinone-like substances, flavins, aromatic amino acids, and dipeptides in SMP and EPS phases were comprehensively investigated and these electron shuttles were significantly affected by Fe_3_O_4_. Dipeptides consisting of phenylalanine were widely detected in T-EPS of the Fe_3_O_4_ reactor, indicating a potential different extracellular electron exchange pattern with the addition of conductive materials.

## Introduction

Interspecies electron transfer between acidogenic bacteria and methanogens plays an important role in methanogenesis. Direct interspecies electron transfer (DIET) has been proposed to be a pathway for syntrophic methane (CH_4_) production (Morita et al., 2011). Syntrophic partners were considered to be able to transfer electron to each other via pili or outer membrane cytochrome directly (Lovley, 2011; Rotaru et al., 2014a). Due to the high electron transfer and energy utilization efficiencies (Lovley et al., 2011), DIET is attracting more and more attentions.

Recently, conductive materials have been reported to function as electron conduits for facilitating syntrophic methanogenesis via DIET (Liu et al., 2012). Until now, many conductive materials have been confirmed to be able to promote the performance of methanogenesis, such as increasing of the CH_4_ production rate and chemical oxygen demand (COD) removal rate, and shortening of the lag phase of methanogenesis (Zhao et al., 2015; Yin et al., 2017a). Methanogenesis can be enhanced by ferroferric oxide (Fe_3_O_4_) with the improvement of the extracellular electron transfer activity of the electron transport chain (Yin et al., 2018). Nevertheless, how electron is transported from surfaces of microorganisms to conductive materials remains unknown. Although outer membrane cytochromes might participate in the direct electron transfer among microorganisms (Rotaru et al., 2014b), in some cases, they are not expected to involve in electron transfer via direct contact with insoluble iron-containing minerals. For example, OmcA and MtrC which involved in the metal-reducing pathway of *Shewanella* were not kinetically competent to be responsible for direct contact with minerals since their relevant rate constants of physiological goethite reduction were three orders of magnitude too low than that of total membrane fractions (Ross et al., 2009). Instead, electron shuttles such as flavins have been reported to promote the electron exchange between microorganisms and minerals (Marsili et al., 2008; Ross et al., 2009). In addition, humic substances have also been shown to serve as soluble electron carriers between microorganisms and insoluble electron acceptors (Coates et al., 2002). Therefore, the interaction between conductive materials and electron shuttles deserves further investigation so as to better understand the extracellular electron transfer mechanism.

Soluble microbial products (SMP) and extracellular polymeric substances (EPS) can be produced by microorganisms and mainly comprised of proteins, polysaccharides, and humic substances (Ramesh et al., 2006). EPS are associated with the cell surfaces and can be divided into external layer (loosely bound EPS, L-EPS) and internal layer (tightly bound EPS, T-EPS), which plays an important roles in microbial aggregation and cell surface adhesion. Both SMP and EPS act as the connection media and bridges between solid minerals and microbes (Yin et al., 2015; Zhu et al., 2017). What’s more, SMP and EPS could store electron shuttles such as c-type cytochromes, aromatic amino acids, and quinone substances, which might potentially benefit the extracellular electron transfer process (Watanabe et al., 2009; Lovley, 2017; Ye et al., 2018). Although components and concentration of SMP and EPS could be affected by external conditions (Le and Stuckey, 2017a; Wei et al., 2017), few studies have focused on the effect of conductive materials on the production of SMP and EPS. Besides, due to the limitation of detecting techniques, previous studies of the components of SMP and EPS were only confined to proteins, polysaccharides, and humic substances. Specifically, little is known about the detailed components of electron shuttles in SMP and EPS, as well as interaction between electron shuttles in SMP and EPS and conductive materials. Herein, better understanding of the effect of conductive materials on concentrations and components of SMP and EPS will contribute to the clarification of the role of conductive materials during CH_4_ production.

In this study, knowledge gap was addressed by investigating the effect of conductive materials on the performance of methanogenesis and the production of SMP and EPS in anaerobic reactors. Both the components and concentrations of SMP and EPS were analyzed. The distribution of potential electron shuttles in SMP and EPS at different anaerobic reaction steps were clarified using mass spectrometry tandem quadrupole time-of-flight mass spectrometer coupled with liquid chromatography system (LC-QTOF-MS). The aim of this study was to provide useful information for the clarification of interactions between conductive materials and SMP and EPS.

## Materials and Methods

### Experimental Setup and Operation

Anaerobic sludge was collected from a lab-scale anaerobic reactor conducted in two 2-L anaerobic sequencing batch reactors (ASBRs) at 35 ± 1°C. The volatile suspended solids (VSSs) of the inoculated sludge was 3.47 ± 0.31 g/L. The control reactor contained inoculated anaerobic sludge and synthetic wastewater, while Fe_3_O_4_ was additionally added as the conductive material in the Fe_3_O_4_ reactor at the optimal concentration of 10 g/L (Yin et al., 2017b). The ASBR reaction cycle consisted of 23 h of anaerobic mixing (including 6 min filling), 54 min of settlement, and 6 min of decanting. The hydraulic retention time was 48 h. Tryptone with 2000 mg/L of COD was fed into synthetic wastewater as the organic substrate. The components of synthetic wastewater and trace elements were similar to the previous study (Yin et al., 2018).

### Batch Experiments

To examine the methanogenic activity of anaerobic sludge in the two reactors, under steady state, batch experiments were conducted. One hundred and twenty-five milliliters aliquots of sludge was taken from the two reactors immediately after effluent discharge, and placed in bottles with 250 mL working volume. Then, 125 mL of synthetic wastewater was added into each bottle and nitrogen gas (N_2_) was used to remove oxygen from the headspace of the bottles for 3 min. The bottles were sealed with rubber stoppers, and placed in an air bath shaker at 170 r/min and 35 ± 1°C. During reaction, liquid and gas samples were periodically collected to analyze concentrations of COD, volatile fatty acids (VFAs), CH_4_, SMP, and EPS, respectively. The experiment was conducted until the amount of CH_4_ production within an hour accounted for less than 10% of the total CH_4_ production.

### Analytical Methods

Chemical oxygen demand and VSS were measured following the standard methods (APHA et al., 2005). CH_4_ and VFAs were determined using gas chromatography (GC-2014, Shimadzu, Japan) according to the method described previously (Yin et al., 2017b). Proteins were measured according to the method described by Lowry et al. (1951). Humic substances were determined and calculated according to Frølund et al. (1995). Polysaccharides were measured by the sulfuric acid-phenol method (Dubois et al., 1951). The modified Gompertz model was applied to calculate the kinetic parameters of CH_4_ production (Zwietering et al., 1990).

Three-dimensional excitation emission matrix (3-D EEM) spectra were obtained using a fluorescence spectrophotometer (Hitachi F-7000, Hitachi, Japan) with an excitation range from 220 to 450 nm and an emission range from 240 to 600 nm both in 5 nm sampling intervals. The spectra were recorded at a scan rate of 12,000 nm/min. To further quantitatively analyze EEM spectra, fluorescence regional integration (FRI) was applied to calculate the contribution proportion of each region in EEM spectra (Chen et al., 2003).

### Detection of Metabolites in SMP and EPS

A 50 mL of sludge suspension was dewatered by centrifugation at 3500 rpm for 10 min. The supernatant was collected as SMP. A heat extraction method was used to extract different EPS fractions according to Li and Yang (2007). For metabolites analysis, EPS and SMP were concentrated by solid phase extraction (SPE). The EPS and SMP samples (50 mL) were pumped through the cartridges (Strata, 200 mg, Phenomenex, United States) for 60 min and the SPE cartridges were dried with N_2_. After being eluted with 5 mL of methanol, the eluted solutions were evaporated to 0.2 mL under a gentle stream of N_2_ with a dry thermo bath (MG-2000, EYELA, Japan). The residues were re-dissolved in 2 mL of ultrapure water for further detection.

After pretreatment, samples were analyzed by an Ion Mobility Q-TOF MS system (Agilent 6560 IM Q-TOF) coupled with an LC system (Agilent 1290 Infinity, United States) (LC-IM-QTOF-MS). The injection volume was 10 μL. The separation of SMP and EPS was performed with a reverse phase column (InertSustain AQ-C18, SHIMADZU, Japan) with a 1:1 mixture of 0.1% formic acid and methanol at a flow rate of 0.3 mL/min as the mobile phase. Q-TOF mode was applied with ion source of Dual AJS electrospray interface (ESI) with mass correction at reference masses of m/z 119.0363 and 966.0070. Electrospray ionization in the negative and positive ion modes was applied to obtain high-resolution mass spectra (m/z 40–1700) of metabolites.

The MS data in QTOF was further analyzed by the Agilent MassHunter Workstation and Profinder software. Metabolites were identified by matching the result with the Metlin metabolism database. Compounds were searched with 5 ppm of search tolerance of precursor ion masses and then were identified with the isotopic distribution similarity above 90%. Principal component analysis (PCA) was conducted to analyze the distinctiveness of specific metabolites using the correlation matrix by the SPSS software.

## Results And Discussion

### Long Term Dosage of Fe_3_O_4_ Promoted Methane Production

**FIGURE 1:**
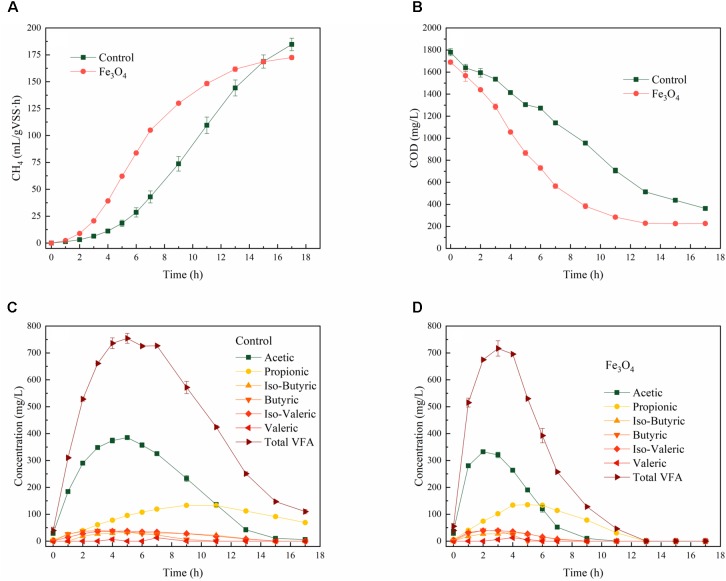
Dynamics of **(A)** COD, **(B)** CH_4_, **(C)** VFAs-control, and **(D)** VFAs-Fe_3_O_4_ within the reaction cycle.

The presence of Fe_3_O_4_ obviously enhanced methanogenesis during the reaction cycle under steady-state conditions (**Figure 1A**). **Supplementary Table S1** shows the dynamic parameters of CH_4_ production fitted by the modified Gompertz model. The lag phase of methanogenesis was shortened by 53.6% (from 4.7 ± 0.6 to 2.2 ± 0.1 h) and the maximum CH_4_ production rate was increased by 23.3% (*R*^2^= 0.999) with the addition of Fe_3_O_4_ (from 17.6 ± 0.7 to 21.7 ± 0.3 mL/L⋅h). This improvement was similar to the result reported by Cruz Viggi et al. (2014) that the supplementation of magnetite enhanced the CH_4_ production rate by 33% from propionate. Consistent with the methanogenesis, the COD degradation (**Figure 1B**) was also induced by Fe_3_O_4_, indicating the rapid conversion of carbon sources from liquid phase to gas phase. As the main intermediate products during the degradation of tryptone, VFAs, especially acetic acid and propionic acid in two reactors, were accumulated at the beginning of the reaction cycle (**Figures 1C,D**). However, total VFAs (calculated as acetic acid) reached the peak concentration earlier and then decreased faster with the addition of Fe_3_O_4_, indicated that conductive materials not only increased CH_4_ production but also promoted hydrolysis and acidification during the reaction cycle. Cruz Viggi et al. (2014) and Jing et al. (2017) also reported that the methanogenesis of propionate was facilitated by magnetite. In addition, the concentration of propionic acid did not decrease until acetic acid was consumed to a certain level (similar concentration to propionic acid). This might be because the standard Gibbs free energy of the conversion of propionate to acetate is positive (ΔG^0′^= 72.7 kJ/mol, 37°C, pH 7), which indicated that the degradation of propionate to acetate cannot occur spontaneously. Therefore, high concentrations of acetic acid would further inhibit the degradation of propionic acid. The presence of Fe_3_O_4_ promoted the consumption of acetic acid and thus facilitated the oxidation of propionic acid (Jing et al., 2017).

### Fe_3_O_4_ Induced the Production of SMP and EPS

**FIGURE 2:**
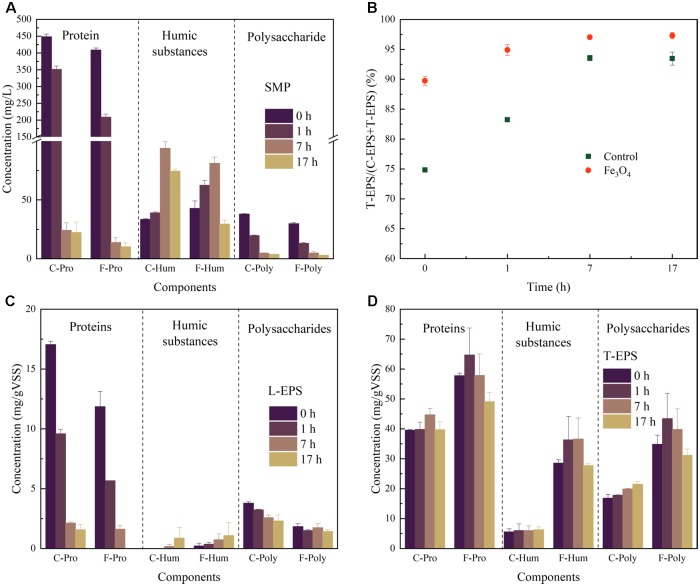
**(A)** Components of SMP, **(B)** the ratio of T-EPS and T-EPS+L-EPS, **(C)** components of L-EPS, and **(D)** components of T-EPS at different time in the control and Fe_3_O_4_ reactor.

The components of SMP during the reaction cycle was analyzed at 0, 1, 7, and 17 h, representing the SMP from the beginning of reaction cycle, the acid fermentation step (VFAs accumulation), the methanogenesis step (with high CH_4_ production rate), and the end of methanogenesis, respectively (**Figure 2A**). A high concentration of proteins at 0 and 1 h was observed which probably due to tryptone in the synthetic wastewater. Therefore, the exact concentration of proteins secreted by microorganisms could not be determined in this study. Nevertheless, protein-based substrates were consumed faster with the addition of Fe_3_O_4_, showing a consistent result with the degradation of COD and production of CH_4_. Humic substances and polysaccharides were detected in both reactors. Humic substances were increased firstly and then decreased after 7 h (the methanogenesis step) of operation, indicating their potential correlation with the methanogenic activity. While polysaccharides with relatively low concentrations (from 2.91 ± 0.08 to 37.96 ± 0.31 mg/L) were decreased during the whole reaction cycle.

As shown in **Figure 2B**, T-EPS contained more proteins, polysaccharides, and humic substances than those of L-EPS. The T-EPS/(T-EPS+L-EPS) ratios of the two reactors increased during the reaction cycle, whereas the reactor with Fe_3_O_4_ had slightly higher percentage (from 89.7 ± 0.8% to 97.3 ± 0.5%) than that of the control reactor (from 74.8 ± 0.3% to 93.5 ± 1.1%). Generally, the differences of components in the L-EPS were not significant between the control and Fe_3_O_4_ reactors (**Figure 2C**). However, the addition of Fe_3_O_4_ considerably promoted the excretion of T-EPS (**Figure 2D**). The highest contents of proteins, humic substances, and polysaccharides in the Fe_3_O_4_ reactor were 64.7 ± 9.0, 36.61 ± 7.0, and 43.4 ± 8.5 mg/g VSS, respectively, and were increased by 44.7, 478.6, and 102.0% from the control reactor. Similar results were reported by Ye et al. (2018) who stated that the addition of red mud (mainly consisted of hematite) enhanced the excretion of EPS during methanogenesis.

Proteins and polysaccharides appeared to play major roles in supporting the bacterial surface adhesion, coaggregation, and system performance (Yin et al., 2015; Le and Stuckey, 2017b). The predominance of proteins in EPS likely indicated the presence of a large amount of exoenzymes (Frølund et al., 1995). Since T-EPS was tightly bound with the cell surface, one explanation of the increasing content of proteins due to the addition of Fe_3_O_4_ might be because more exoenzymes were induced to participate in the extracellular activity. Ye et al. (2018) found that a higher abundance of c-type cytochromes involved in DIET was excreted in the presence of red mud, especially in T-EPS, resulting in the improved CH_4_ production. On the other hand, proteins were also suggested to be responsible for the surface property of the microbial aggregates, such as hydrophobicity and surface charge (Jorand et al., 1998), depending on the characteristics of amino groups on proteins. Compared with proteins, polysaccharides appeared to mediate the cell aggregation through different mechanisms. For example, some gelling moieties (e.g., alginate-like polysaccharide) were proposed to benefit the cell aggregation (Gonzalez-Gil et al., 2015). Besides, polysaccharides contained long backbone with active side chains such as uronic acids and acetyl amino sugars, which were able to provide plenty of binding sites for microorganisms (Li et al., 2012). Due to the low energy produced during methanogenesis and the lack of carrier molecules involved in the release of polysaccharides, methanogens were suggested to be unlikely play a key role in the production of polysaccharides (Morgan et al., 1990). Therefore, other microorganisms such as fermentative bacteria and acidogenic bacteria might be main contributors for the promoted polysaccharides excretion with the addition of Fe_3_O_4_.

Humic substances which increased the most in this study, were believed to play an important role for fermentative bacteria and anaerobic respiratory bacteria (Coates et al., 2002). The content of humic substances was reported to be positively correlated with the electron-accepting and donating capacity, and it was probably due to the existence of some effective electron-exchanging substances such as quinones (Jiang and Kappler, 2008; Ye et al., 2018). Ho and Ho (2012) demonstrated that VFAs consumption and methanogenesis during anaerobic treatment were stimulated by the addition of humic substances. What’s more, moieties in humic substances were shown to act as soluble electron carriers between microorganisms and insoluble electron acceptors (Lovley and Bluntharris, 1999). Therefore, it could be highly suspected that the higher contents of humic substances induced by Fe_3_O_4_ might participate in extracellular electron transfer and be partly responsible for the high efficiency of methanogenesis and VFAs degradation.

### EEM Fluorescence Spectra Analysis

**FIGURE 3:**
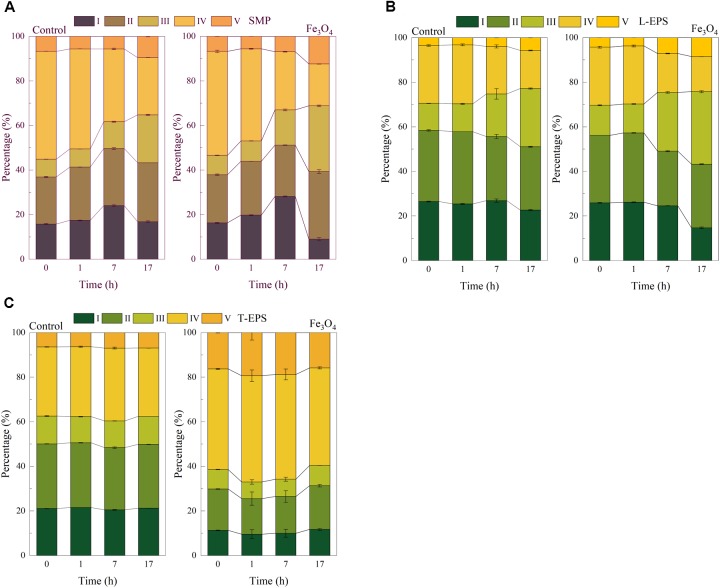
Percentage of each region of **(A)** SMP, **(B)** L-EPS, and **(C)** T-EPS at different time in the control and Fe_3_O_4_ reactor.

The EEM fluorescence spectra were applied to further distinguish the organic compounds in SMP and EPS. Compared with humic substances and proteins, the intensity of EEM fluorescence spectrum of polysaccharides was much weaker and could be neglected (Sheng and Yu, 2006). Therefore, the fluorescence signals of SMP and EPS were mainly attributed to humic substances and proteins. As shown in **Supplementary Figure S1**, several peaks were found and located in different regions. **Figure 3A** shows the percentage of each region in SMP. At the beginning and the acid fermentation step (the 0 and 1 h), soluble microbial by-products and aromatic protein-like substances (region IV) which were located at the excitation/emission wavelengths (Ex/Em) of 250–360/280–380 nm were predominant in the two reactors, accounting for more than 40% of SMP. Then soluble microbial by-products and aromatic protein-like substances were decreased afterward and accounted for 25.6 and 18.8% in the control reactor and the Fe_3_O_4_ reactor at 17 h, respectively. Since soluble microbial by-products were regarded as biodegradable materials (Jia et al., 2013), this result indicated that substances located at region IV might be consumed during the reaction cycle. Tyrosine-like substances (region I, located at Ex/Em of 220–250/280–330 nm), another biodegradable substance, were increased during the acid fermentation and methanogenesis step, and then decreased afterward. One possible explanation might be that microorganisms tended to produce tyrosine-like substances at rich organic carbon-conditions and these biodegradable substances could be consumed at organic carbon limited conditions to maintain their survival. On the contrary, tryptophan-like substances (region II, located at Ex/Em of 220–250/330–380), fulvic acid-like substances (region III, located at Ex/Em of 220–250/380–480 nm), and humic acid-like substances (region V, located at Ex/Em of 250–420/380–520 nm), which are regarded as non-biodegradable substances (Jia et al., 2013), were all accumulated during the whole reaction cycle. The percentages of these substances at the end of methanogenesis (at 17 h) were increased with the addition of Fe_3_O_4_ (increased by 3.8, 8.0 and 2.8% of regions II, III, and V, respectively), indicating that the dosage of Fe_3_O_4_ facilitated the excretion of non-biodegradable substances in SMP.

**Figure 3B** shows the distribution of five regions in L-EPS. Tyrosine-like, tryptophan-like, and soluble microbial by-products substances (regions I, II, and IV, respectively) were predominant at 0 and 1 h (**Supplementary Figure S2**). Similarly, biodegradable substances (regions I and IV) were then decreased and non-biodegradable substances (regions III and V) were accumulated. Compared with the control reactor, the distribution of fulvic acid-like and humic acid-like substances were increased by 6.4 and 2.8% in the presence of Fe_3_O_4_, respectively, but the effect on tryptophan-likes substances (region II) was not significant.

The addition of Fe_3_O_4_ significantly affected the distribution of T-EPS (**Figure 3C**). As shown in **Supplementary Figure S3**, the intensity of peaks in regions IV and V were higher than that of the control reactor. The result of FRI showed that the distribution of soluble microbial by-products and aromatic protein-like substances (region IV) was accounted for 43.5 to 47.6%, which was increased by 13.2–16.2% in the Fe_3_O_4_ reactor. Similarly, the percentages of humic acid-like substances (region V) were also promoted by Fe_3_O_4_, with the distribution accounting for 15.8–19.4% in the Fe_3_O_4_ reactor and 5.6–12.4% in the control reactor. However, the percentages of tyrosine-like (region I) and tryptophan-like substances (region II) were decreased with the dosage of Fe_3_O_4_. Another distinct result was that the change of these substances was relatively minor during the reaction cycle, compared with that in SMP and L-EPS. One possibility was that the potential roles of these substances in T-EPS might be different from SMP and L-EPS. One the one hand, Lovley (2017) proposed that aromatic amino acid (e.g., tyrosine and tryptophan) were key elements for e-pili conductivity and the overlapping π-π orbitals of aromatic amino acids might possibly be responsible for the metallic-like conductivity. On the other hand, as mentioned above, humic substances were correlated with the electron accepting and donating capacity, indicating that they could participate in the extracellular electron transfer (Ye et al., 2018). Therefore, substances located at five regions might all contribute to the extracellular electron exchange potentially. Combined with the above results, it could be further suspected that the contribution of these substances on extracellular electron exchange might be different in the two reactors. In the control reactor, tyrosine-like substances (region I), tryptophan-like substances (region II), and other aromatic protein-like substances (region IV) might be main potential electron shuttles. While in the presence of Fe_3_O_4_, aromatic protein-like substances (region IV) and humic acid-like substances (region V) might be the dominant contributors for the extracellular electron transfer.

### Distribution of Electron Shuttles Detected by LC-IM-QTOF-MS

The LC-IM-QTOF-MS was further applied to obtain insights into specific components in SMP and EPS. Due to the rich diversity of metabolites in SMP and EPS, and high possibilities of some metabolites with same masses but various chemical structures, the existence of compounds matched by database could not be accurately confirmed in this study. Therefore, compounds with different optical activity (e.g., D-tyrosine, L-tyrosine, and DL-tyrosine) and isomeric compounds (e.g., ribose and xylose, arginyl-leucine, and leucyl-arginine) which could not be distinguished based on matched results were reported as a mixture (Tipthara et al., 2017). Herein, only amino acids, dipeptides, monosaccharide, quinones, and their derivatives were discussed. It should be noted that there are some possibilities that other kinds of metabolites with same masses might also be matched by the database.

**FIGURE 4:**
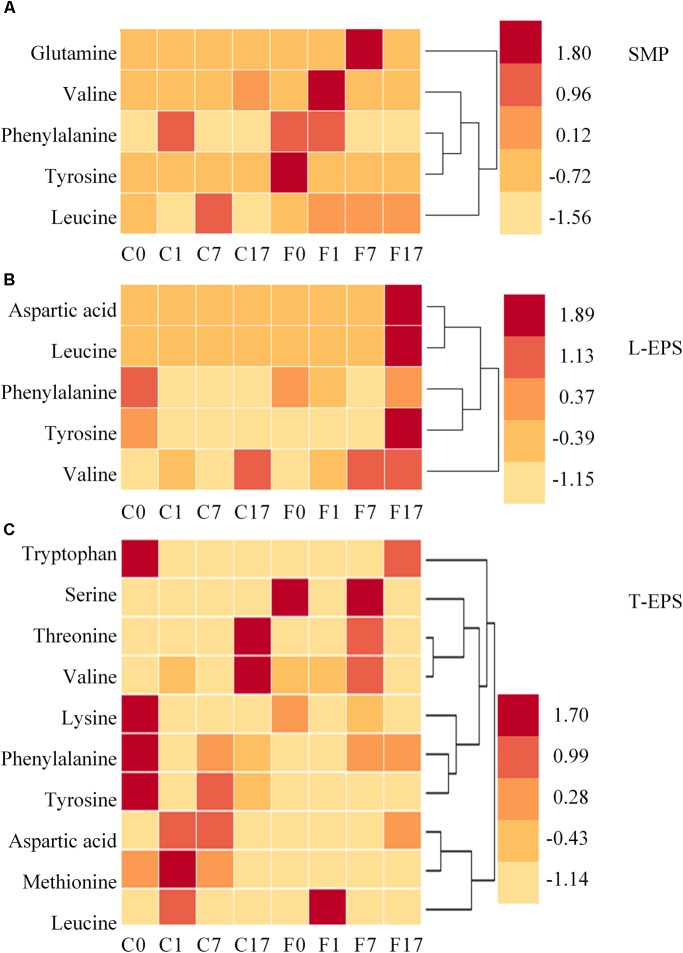
Distribution of amino acids in panel **(A)** SMP phase, **(B)** L-EPS phase, and **(C)** T-EPS phase at different steps in the control and Fe_3_O_4_ reactors. C0, C1, C7, and C17 refer to samples collected from the control reactor at 0, 1, 7, and 17 h. F0, F1, F7, and F17 refer to samples collected from the Fe_3_O_4_ reactor at 0, 1, 7, and 17 h.

Despite above limitation, the result provides novel perspective of the effect of conductive material on SMP and EPS. **Table 1** shows matched information of amino acids detected in SMP and EPS. To evaluate the variation of amino acids in different samples, their peak intensities were standardized by *z*-score and are shown in **Figure 4**. Amino acids were more diverse in T-EPS than in SMP and L-EPS. Valine, phenylalanine, tyrosine, and leucine were detected in all phases, indicating their wide existence in extracellular environment. The distribution of amino acids showed significant difference between the control and Fe_3_O_4_ reactors. For example, the intensities of most amino acids in SMP were higher at different steps with the addition of Fe_3_O_4_ (**Figure 4A**). A similar result was found in L-EPS samples despite the fact that the highest intensities were obtained at the end of methanogenesis in the Fe_3_O_4_ reactor (**Figure 4B**). In T-EPS of the control reactor, different kinds of amino acids were accumulated at various steps (**Figure 4C**). On the contrary, less amino acids were found at 0 and 1 h in the Fe_3_O_4_ reactor. Since amino acids were responsible for multifunction, the change of amino acids might be due to many reasons. Firstly, amino acids might be used as carbon source by microorganisms (Zhao et al., 2018). Secondly, the production or degradation of peptides and proteins might also cause the consumption or accumulation of amino acids. Thirdly, amino acids were precursors of biosynthesis of nucleotide sugar, phospholipids, and peptidoglycan (Xu and Liu, 2011; Zhao et al., 2018), and these biosynthesis processes could further lead to the consumption of amino acids. From the aspect of extracellular electron transfer, amino acids with aromatic ring (phenylalanine, tryptophan, and tyrosine) might be responsible for the metallic-like conductivity and extracellular electron exchange (Lovley, 2017). The highly different changing pattern of amino acids between the control and Fe_3_O_4_ reactors suggested that the biological metabolism was affected by the conductive material.

**Table 1:** Matched information of amino acids detected in SMP and EPS.

Name	Formula	Mass (avg)	Retention time (avg)	Score (max)	Source	Phase	Peak intensity							
							C0	C1	C7	C17	F0	F1	F7	F17
Glutamine	C_5_ H_10_ N_2_ O_3_	146.07	8.23	90.3	Pos	SMP	0	0	0	0	0	0	42998	0
Valine	C_5_ H_11_ N O_2_	117.08	0.98	98.9	Neg	SMP	0	0	0	16279	0	34891	0	6690
Leucine	C_6_ H_13_ N O_2_	131.10	1.7	99.4	Pos	SMP	135029	0	253935	0	123748	190632	216974	205538
Phenylalanine	C_9_ H_11_ N O_2_	165.08	2.74	99.7	Pos	SMP	0	2059396	0	0	2262207	1843377	0	0
Tyrosine	C_9_ H_11_ N O_3_	181.07	11.45	90.4	Neg	SMP	0	0	0	0	128780	7636	0	0
Aspartic acid	C_4_ H_7_ N O_4_	133.04	0.92	91.2	Pos	L-EPS	0	0	0	0	0	0	0	7803
Leucine	C_6_ H_13_ N O_2_	131.09	1.65	99.4	Pos	L-EPS	0	0	0	0	0	0	0	180147
Phenylalanine	C_9_ H_11_ N O_2_	165.08	2.77	99.9	Pos	L-EPS	199231	51904	0	44191	163562	56393	0	156062
Tyrosine	C_9_ H_11_ N O_3_	181.07	1.54	99.4	Pos	L-EPS	60856	0	0	0	0	0	0	175447
Valine	C_5_ H_11_ N O_2_	117.08	2.45	99.1	Pos	L-EPS	0	43652	0	96693	0	39941	100050	99032
Tryptophan	C_11_ H_12_ N_2_ O_2_	204.09	5.2	99.4	Neg	T-EPS	2119669	0	0	0	0	0	0	1772004
Serine	C_3_ H_7_ N O_3_	105.04	5.55	91.2	Pos	T-EPS	0	0	0	0	21007	0	21395	0
Aspartic acid	C_4_ H_7_ N O_4_	133.04	5.17	97.9	Pos	T-EPS	0	43582	37981	0	0	0	0	27129
Threonine	C_4_ H_9_ N O_3_	119.06	4.46	95.8	Pos	T-EPS	0	0	0	48244	0	0	32890	0
Valine	C_5_ H_11_ N O_2_	117.08	1.03	99.8	Pos	T-EPS	73720	188770	0	543238	161966	151458	527631	101798
Methionine	C_5_ H_11_ N O_2_ S	149.05	3.92	99.2	Pos	T-EPS	16631	44991	17174	0	0	0	0	0
Leucine	C_6_ H_13_ N O_2_	131.09	1.7	98.8	Pos	T-EPS	0	153960	0	0	0	281035	0	0
Lysine	C_6_ H_14_ N_2_ O_2_	146.11	0.78	99.2	Pos	T-EPS	240018	0	0	0	88957	0	63249	0
Phenylalanine	C_9_ H_11_ N O_2_	165.08	3.78	99.6	Neg	T-EPS	1406037	0	667810	433314	0	0	800801	801007
Tyrosine	C_9_ H_11_ N O_3_	181.07	1.53	99.3	Pos	T-EPS	3458894	352618	2136986	974609	0	73564	82132	65503

*avg, average; max, maximum; Pos, positive mode; Neg, negative mode.*

**FIGURE 5:**
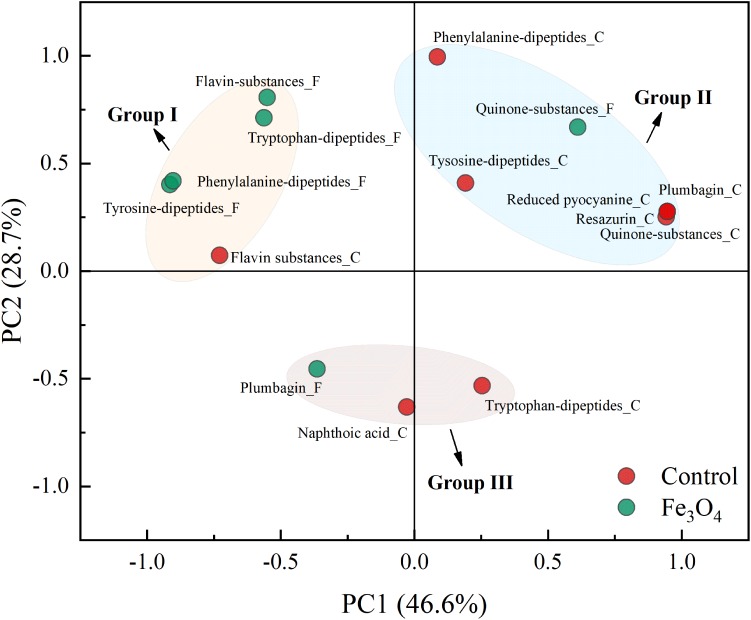
Principal component analysis of dipeptides, quinone substances, and other potential electron shuttles in SMP and EPS phases.

Apart from amino acids, dipeptides, which were derived from the dehydration synthesis of two amino acids were also investigated. **Supplementary Figure S4A** shows the heat map of dipeptides detected in SMP and a total of 42 dipeptides were detected. Specifically, dipeptides with aromatic ring (e.g., diphenylalanine and phenylalanyl-tryptophan) could form the peptide network to serve as the electron transfer mediator (Amdursky et al., 2010; Amdursky, 2013). **Supplementary Figure S5** shows the distributed variation of aromatic dipeptides between the two reactors. Among dipeptides found in SMP, 9 of them contained aromatic ring (consisting of phenylalanine, tryptophan, or tyrosine), accounting for 21.4% of total number of dipeptides (**Table 2**). Tyrosine was the main component of aromatic dipeptides. The distribution of aromatic dipeptides showed significant difference between the control and Fe_3_O_4_ reactors. Five of aromatic dipeptides reached the highest intensity in the control reactor and most of them were obtained at the fermentation step. However, other 4 aromatic dipeptides reached the highest intensity in the Fe_3_O_4_ reactor and they were accumulated mainly at the beginning of the reaction cycle and the end of methanogenesis. A total of 21 dipeptides were found in L-EPS and 7 of them were aromatic dipeptides (**Supplementary Figure S4B**). Compared with SMP, the percentage of aromatic dipeptides in L-EPS was slightly increased (33.3%) and most of these aromatic dipeptides reached to the highest intensity in the control reactor, rather than in the Fe_3_O_4_ reactor. Furthermore, the highest numbers of dipeptides (68) as well as aromatic dipeptides (31) were obtained in T-EPS (**Supplementary Figure S4C**). This result indicated that 45.6% of dipeptides in T-EPS contained aromatic ring which could potentially benefit the extracellular conductivity. More than 50% of aromatic dipeptides consisted of phenylalanine rather than tyrosine, showing an obvious difference to SMP. Moreover, the highest intensities of a large part of aromatic dipeptides were found in the Fe_3_O_4_ reactor (67.7%), including large proportion of aromatic dipeptides consisting of phenylalanine and tyrosine. Phenylalanine and tyrosine were components of PilA and were believed to play an important role in electron transport along the pili (Tan et al., 2016). On the contrary, tryptophan based-aromatic dipeptides were mainly accumulated in the control reactor. Tryptophan could facilitate faster electron transport than phenylalanine and tyrosine in dipeptide networks, but there was no tryptophan in the native PilA (Amdursky, 2013; Tan et al., 2016). In order to confirm the distinctiveness of aromatic dipeptides in two reactors, PCA analysis was conducted based on the total intensity of aromatic dipeptides in SMP and EPS phases. As shown in **Figure 5**, tryptophan, phenylalanine, and tyrosine-based dipeptides in the Fe_3_O_4_ reactor were clustered closely in group I and were separated far from aromatic dipeptides in the control reactor. In addition, tryptophan-based dipeptides in the control reactor were clustered in group III, revealing a significant difference from phenylalanine and tyrosine-based dipeptides (group II). This cluster pattern further confirmed that the distribution of aromatic dipeptides was distinctly affected by dosing Fe_3_O_4_. Future investigation for the quantification of dipeptides in anaerobic sludge is needed to clarify their potential roles in extracellular electron transport.

**Table 2:** Detected result of dipeptides in SMP and EPS.

Phase	Number of total dipeptides	Number of dipeptides with aromatic ring	Percentage of dipeptides with aromatic ring (%)	Number of dipeptides contained phenylalanine	Number of dipeptides contained tryptophan	Number of dipeptides contained tyrosine	Number of aromatic dipeptides with the highest intensity	
							Control reactor	Fe_3_O_4_ reactor
SMP	42	9	21.4	2	1	6	5	4
L-EPS	21	7	33.3	3	2	2	5	2
T-EPS	68	31	45.6	16	8	9	10	21

**FIGURE 6:**
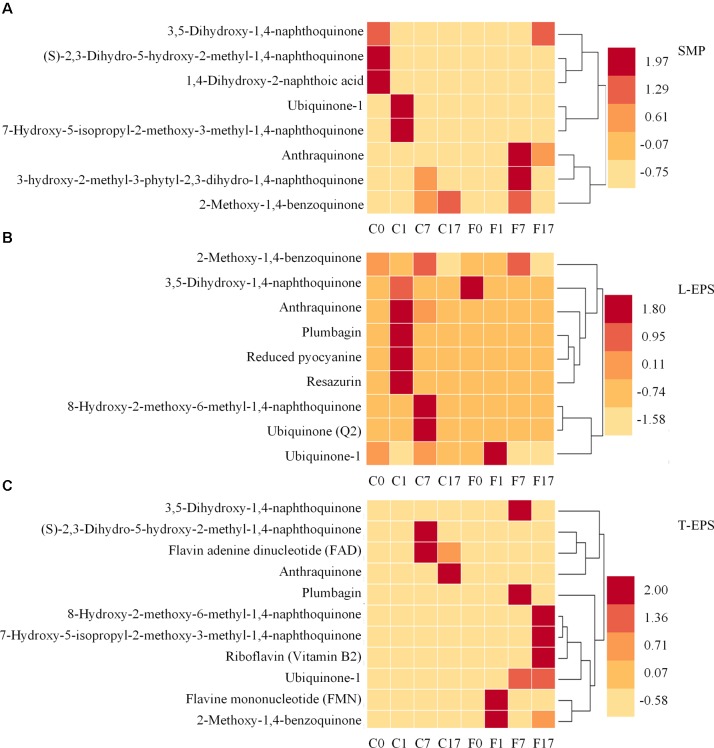
Distribution of quinone substances and other potential electron shuttles in **(A)** SMP phase, **(B)** L-EPS phase, and **(C)** T-EPS phase at different steps in the control and Fe_3_O_4_ reactors. C0, C1, C7, and C17 refer to samples collected from the control reactor at 0, 1, 7, and 17 h. F0, F1, F7, and F17 refer to samples collected from the Fe_3_O_4_ reactor at 0, 1, 7, and 17 h.

As mentioned above, quinone substances were important moieties of humic substances and were considered to serve as electron shuttles (Watanabe et al., 2009). Therefore, to further investigate the effect of Fe_3_O_4_ on electron shuttles in SMP and EPS, quinone substances and their derivatives, and other potential electron shuttles were further analyzed. **Figure 6** shows the distribution of quinone substances and other potential electron shuttles in different phases and reactors. In SMP (**Figure 6A**), ubiquinone, anthraquinone, and derivatives of naphthoic acid, naphthoquinone, and benzoquinone were detected. The existence of quinone groups enabled these components to act as redox mediators potentially (Newman and Kolter, 2000; Kang and Choi, 2008; Watanabe et al., 2009). Ubiquinone playing an important role in the respiratory electron transport chain, was also detected in anaerobic sludge samples by Tipthara et al. (2017). More potential electron shuttles were found in the control reactor at different steps, suggesting that Fe_3_O_4_ not only affected the distribution of amino acids and dipeptides but also quinone substances. In L-EPS (**Figure 6B**), apart from derivatives of quinone substances, plumbagin, reduced pyocyanine and resazurin which were reported to benefit the extracellular electron transport, were also detected (Rau et al., 2002; Hernandez et al., 2004; Watanabe et al., 2009). Flavins, another kind of electron shuttles, were further detected in T-EPS (**Figure 6C**). Flavin adenine dinucleotide (FAD) reached a high intensity in the control reactor, whereas riboflavin (Vitamin B2) and flavin mononucleotide (FMN) were only detected in the Fe_3_O_4_ reactor. Flavins were reported to play crucial roles in electron transfer (Zhu et al., 2017). For instance, it has been shown that outer-membrane c-type cytochromes of *Shewanella oneidensis* was not responsible for electron transfer via direct contact with insoluble iron minerals, whereas the addition of flavins supported the connection between *Shewanella oneidensis* and insoluble iron minerals at sufficient rates (Ross et al., 2009). Moreover, the accumulation of flavins was reported to facilitate the electron transfer rate between biofilms and electrode by 370% (Marsili et al., 2008). More than 80% of these potential electron shuttles were detected in the Fe_3_O_4_ reactor, indicating that conductive materials significantly affected the excretion of electron shuttles in T-EPS. The distinctiveness of these potential electron shuttles was also evaluated by the PCA analysis. As shown in **Figure 5**, Flavin substances in the two reactors were clustered in group I, while quinone substances were clustered in group II, suggesting a distinct distributed variation between flavin substances and quinone substances. In addition, plumbagin, reduced pyocyanine, resazurin, and quinone substances in the control reactor were clustered closely in group II, implying their similar roles in microbial interaction potentially. In contrast, plumbagin substances with the addition of Fe_3_O_4_ were clustered in group III. Although the extracellular electron transfer can be divided into direct electron transfer (via outer membrane cytochrome or pili) and indirect electron transfer mode (via electron shuttles), the interaction between conductive materials and these electron transfer mediators is still unclear. Since Fe-humic acid complex could be formed to function as a solid phase electron mediator (Zhang et al., 2014), it is reasonable to suspect that electron shuttles in SMP and EPS might participate in the electron exchange between microorganisms and conductive materials.

**Supplementary Figure S6** shows the distribution of monosaccharides, disaccharides, and their derivatives. Because monosaccharides with the same carbon atom were isomeric compounds and could not be distinguished by database matching, the result here was reported as molecular formula. Tetrose, derivatives of pentose, and hexose distributing at various steps were detected in SMP and L-EPS. However, no derivatives of pentose and hexose were found in T-EPS. Instead, disaccharide was detected and enriched at the methanogenesis step in the Fe_3_O_4_ reactor. Although the electron exchange ability of polysaccharides has been rarely discussed, other potential roles of polysaccharides (e.g., cell aggregation) might still affect the performance of anaerobic sludge (Li et al., 2012). Since monosaccharides and disaccharides which serve as the precursor of polysaccharides were affected by the presence of Fe_3_O_4_, it should be suggested that the component of polysaccharides might also be influenced by conductive materials. More investigations are needed to further examine their possible functions.

## Implications

This study provided valuable insights into the interaction between conductive materials and SMP and EPS. A long term dosage of Fe_3_O_4_ not only facilitated the methanogenesis with a 23.3% increase but also promoted the T-EPS production. Concentration of proteins, humic substances, and polysaccharide were induced by Fe_3_O_4_, suggesting a different extracellular metabolism with the addition of conductive materials. Potential electron transfer mediators in SMP and EPS were comprehensively investigated and their distributions were significantly affected by Fe_3_O_4_. Humic substances, flavins, and dipeptides might participate in the extracellular electron exchange among microorganisms and insoluble conductive materials, and consequently increased the extracellular electron transfer efficiency. These results allow for a better understanding of the extracellular microbial products in anaerobic reactors, and biogeochemical interaction between conductive materials and redox mediators excreted by microorganisms. Regulating the production of macromolecule through the supplement of conductive materials may be a potential approach to achieve the optimization of extracellular electron transfer pathway during anaerobic treatment. For practical application, with feasible magnetic separation and recycling, the supplement of Fe_3_O_4_ can be applied to the anaerobic reactor to retain a high biomass density, enhance the efficiency of anaerobic treatment, and promote the methane biofuel production.

## Author Contributions

GW designed the experiments and revised the manuscript. QY constructed the experiments and wrote the manuscript. KH constructed the QTOF analysis. SE, XZ, and HH revised and modified the manuscript.

## Conflict of Interest Statement

The authors declare that the research was conducted in the absence of any commercial or financial relationships that could be construed as a potential conflict of interest.
