# Supplementary material for: Ferroferric Oxide Significantly Affected Production of Soluble Microbial Products and Extracellular Polymeric Substances in Anaerobic Methanogenesis Reactors
Source: Front Microbiol. 2018 Oct 9;9:2376. doi: 10.3389/fmicb.2018.02376 (PMC6189335; doi:10.3389/fmicb.2018.02376)
Supplement: Supplementary file 1 [file Data_Sheet_1.docx]

**Supplementary Materials**

**Table S1.** Dynamic parameters calculated by the modified Gompertz model

| Group | Maximum CH_4_ yield (mL/L) | Lag phase (h) | Maximum CH_4_ production rate (mL/L·h) | R^2^ |
| --- | --- | --- | --- | --- |
| Control | 231.4±3.0 | 4.7±0.6 | 17.6±0.7 | 0.999 |
| Fe_3_O_4_ | 173.6±2.7 | 2.2±0.1 | 21.7±0.3 | 0.999 |


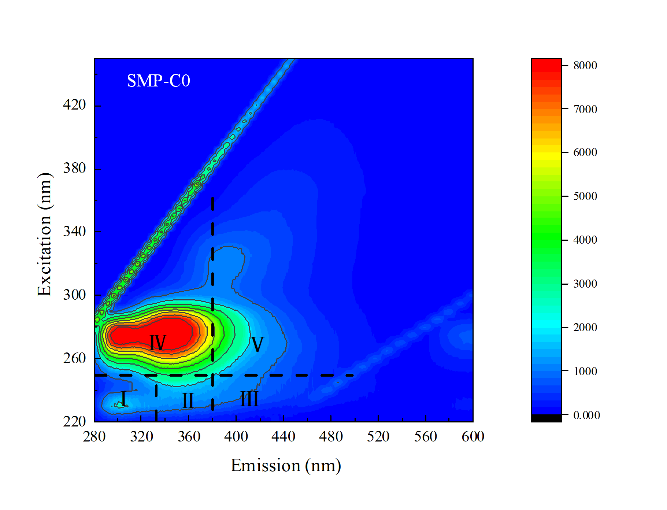

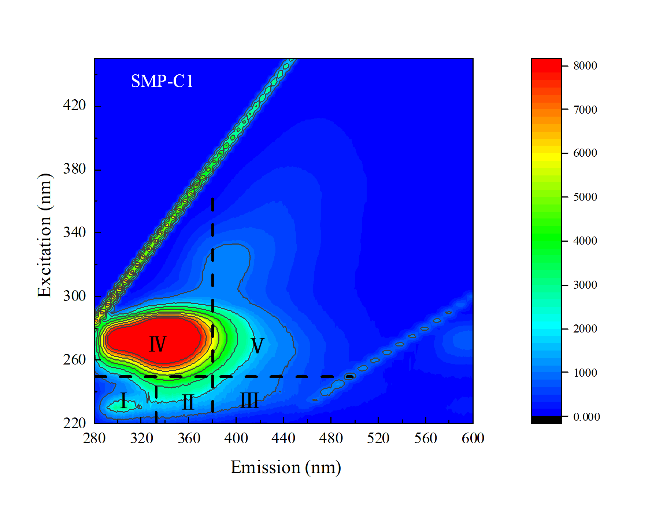


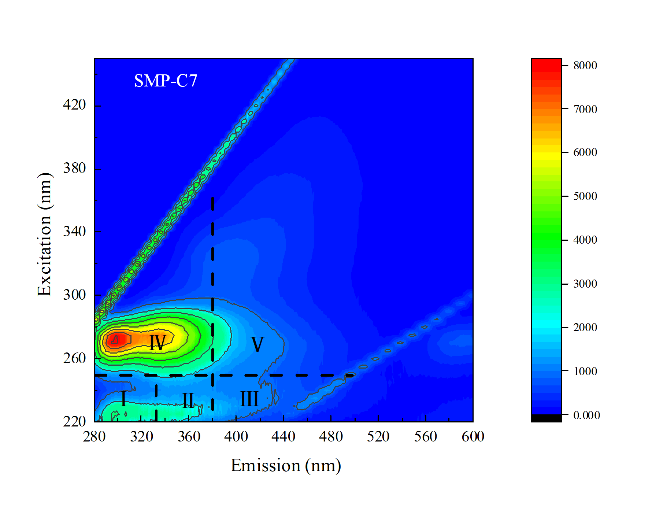

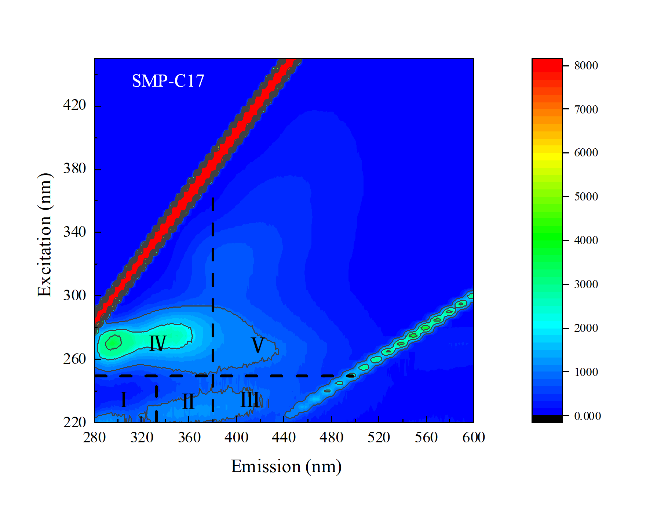


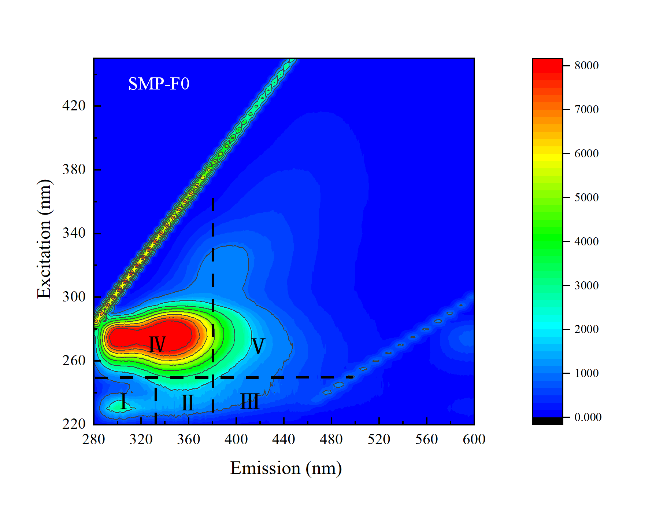

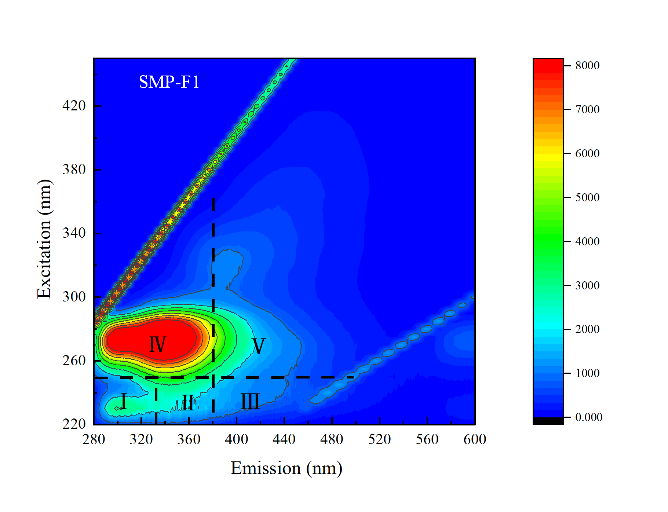


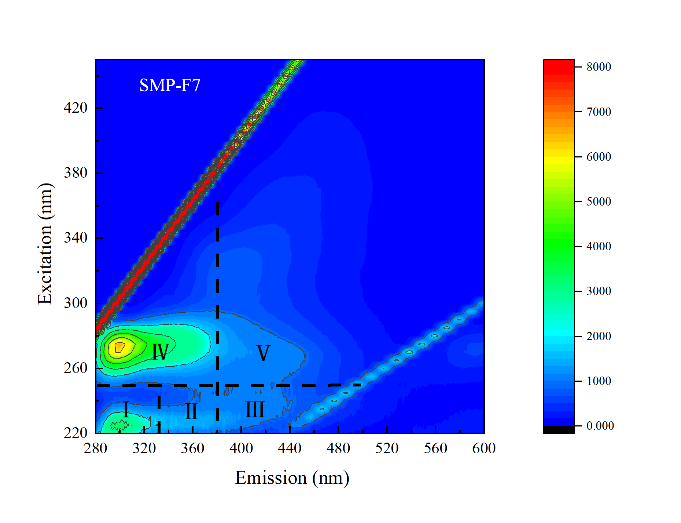

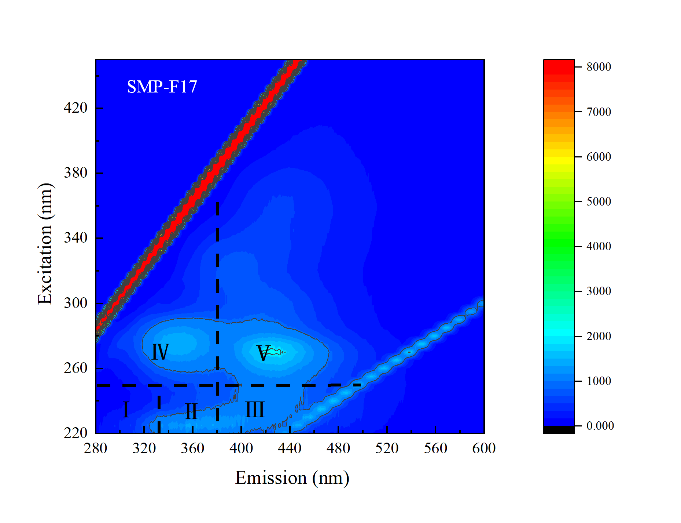


**Figure S1.** EEM fluorescence spectra result of SMP in the control and Fe_3_O_4_ reactors.


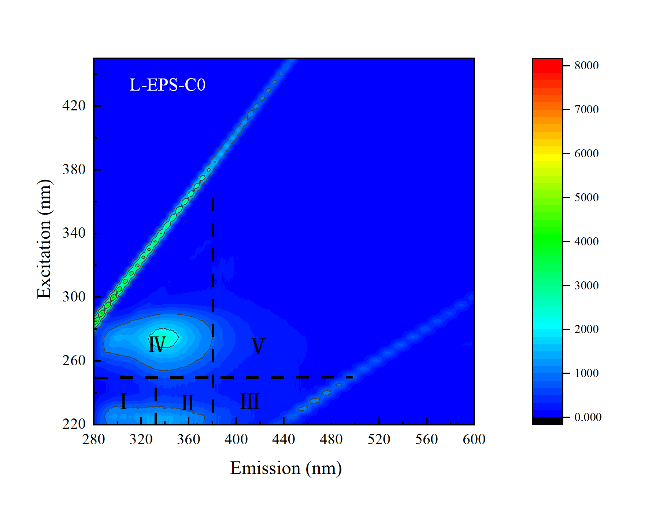

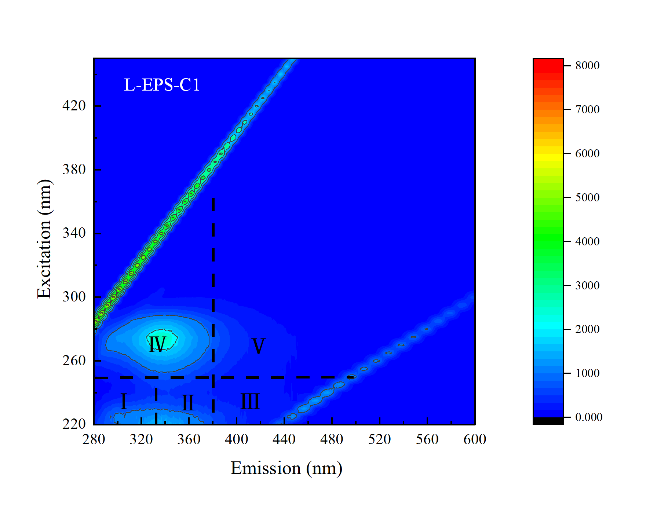


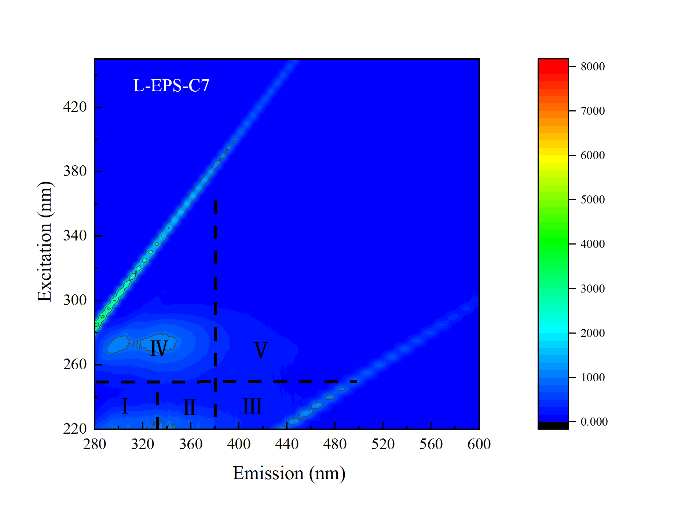

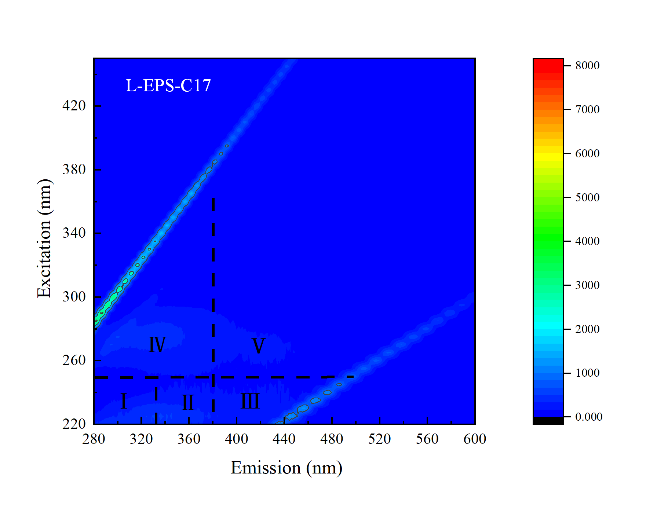


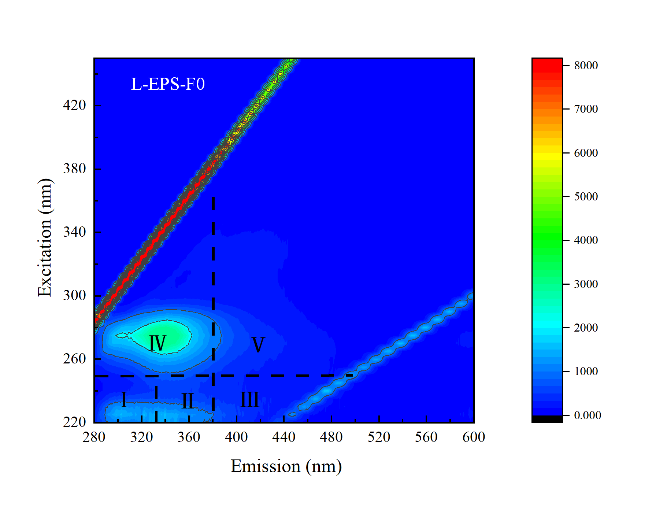

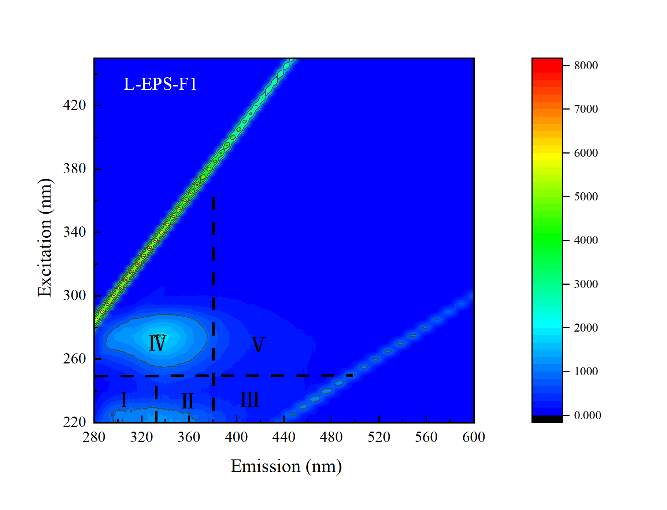


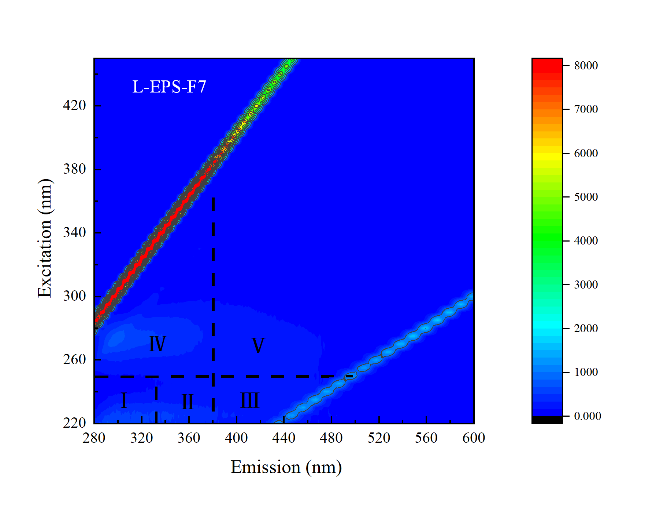

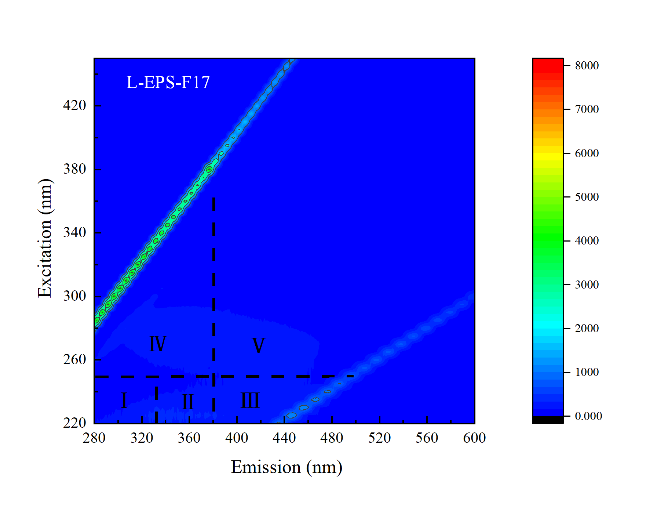


**Figure S2.** EEM fluorescence spectra result of L-EPS in the control and Fe_3_O_4_ reactors.


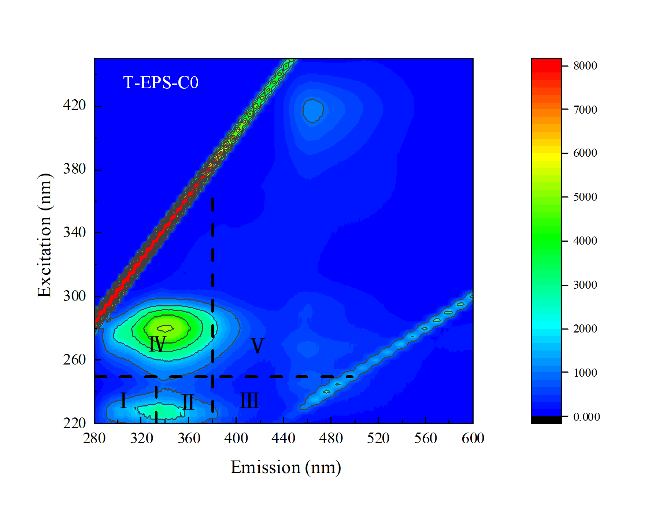

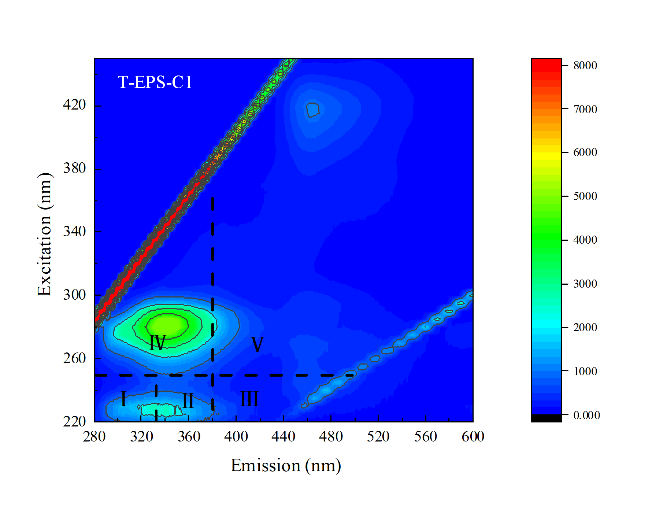


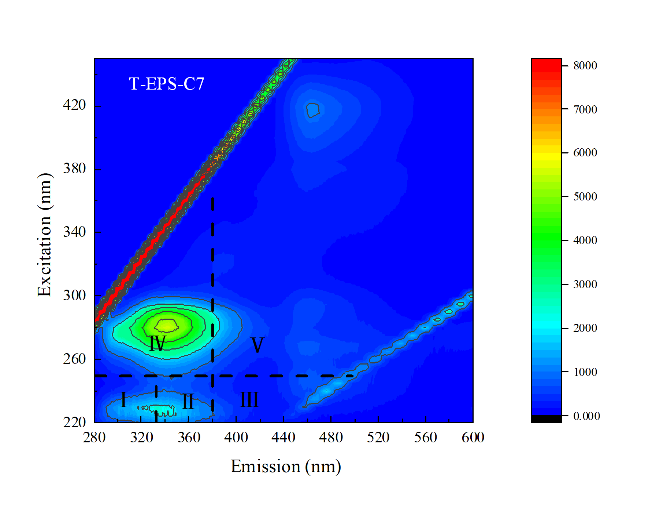

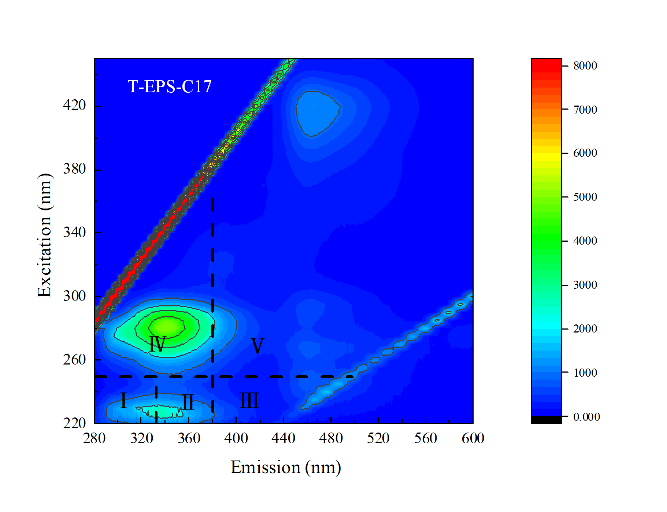


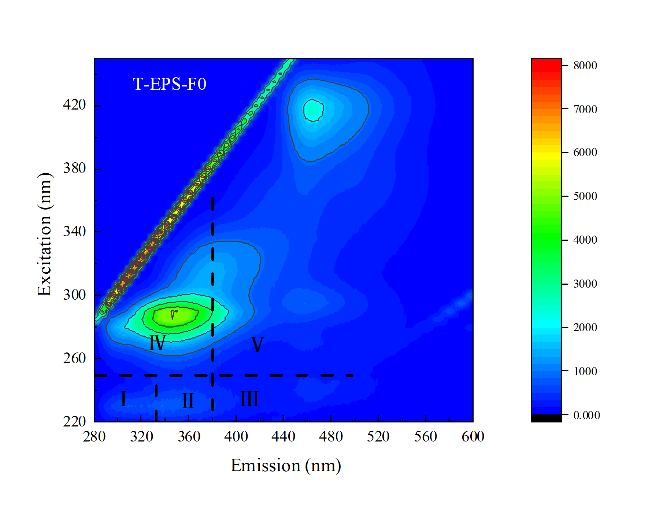

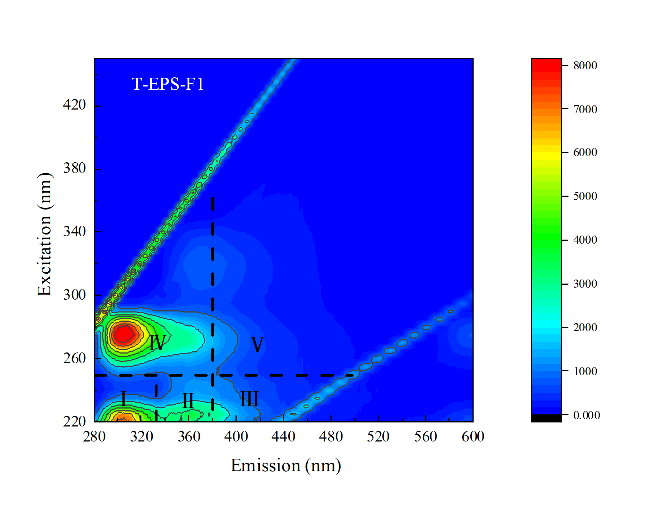


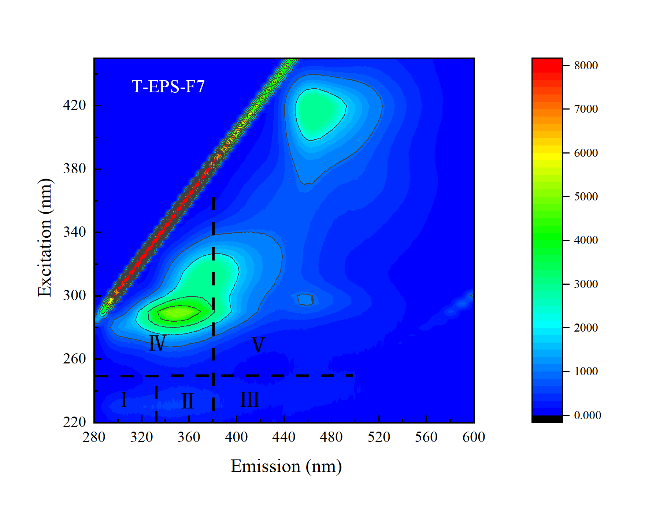

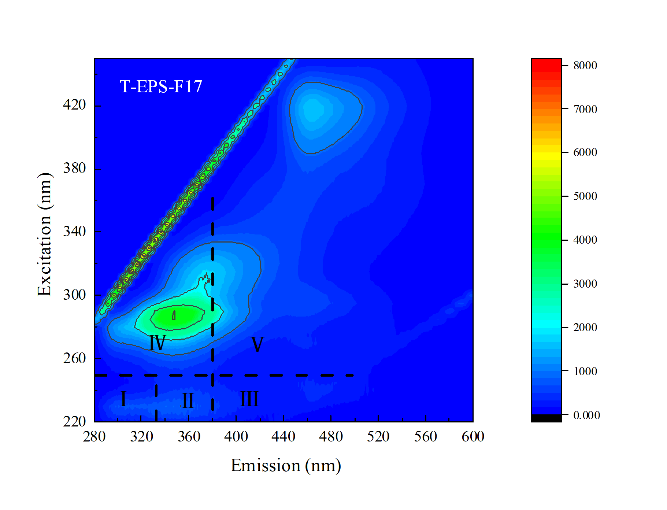


**Figure S3**. EEM fluorescence spectra result of T-EPS in the control and Fe_3_O_4_ reactors.


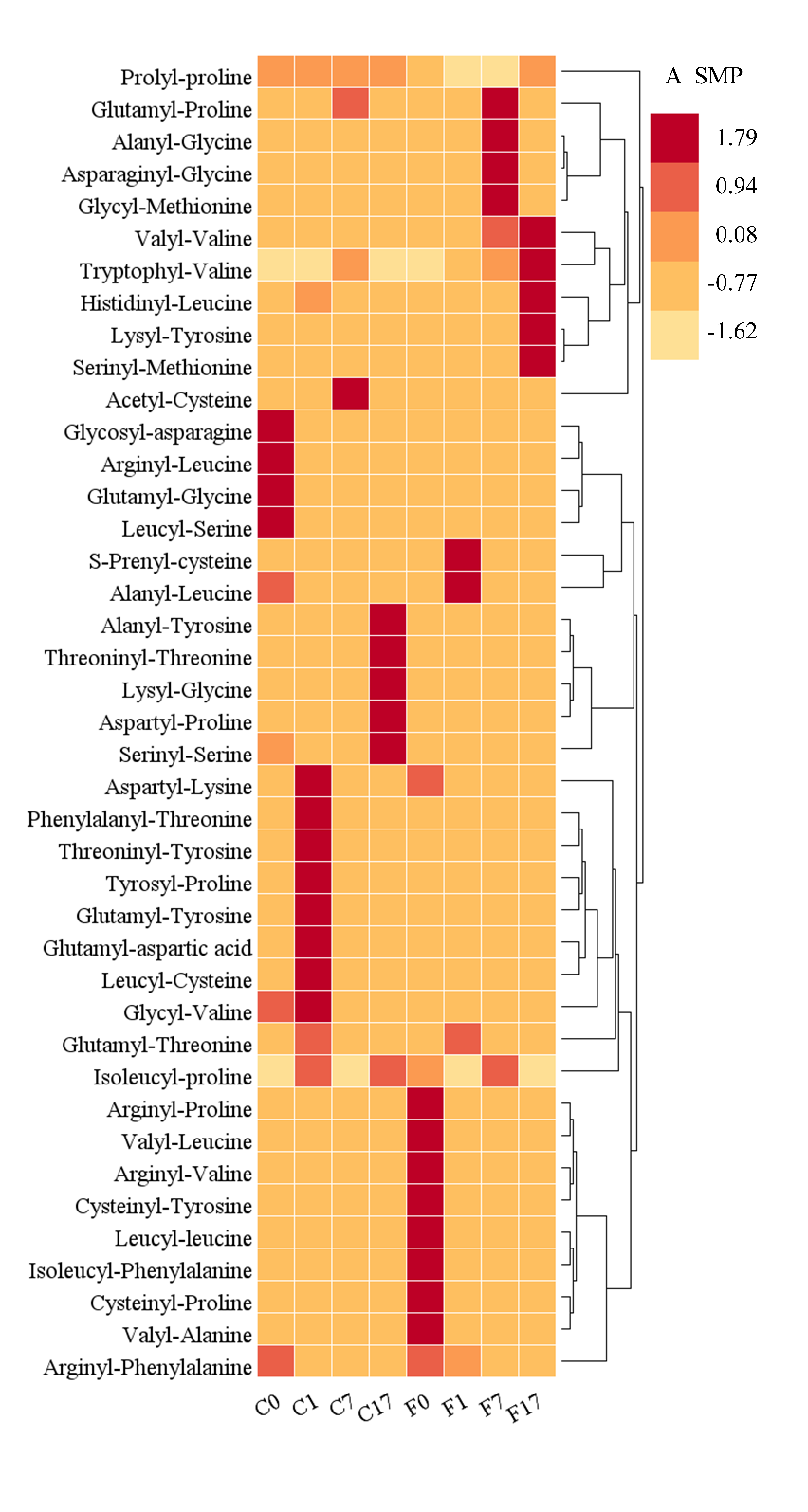


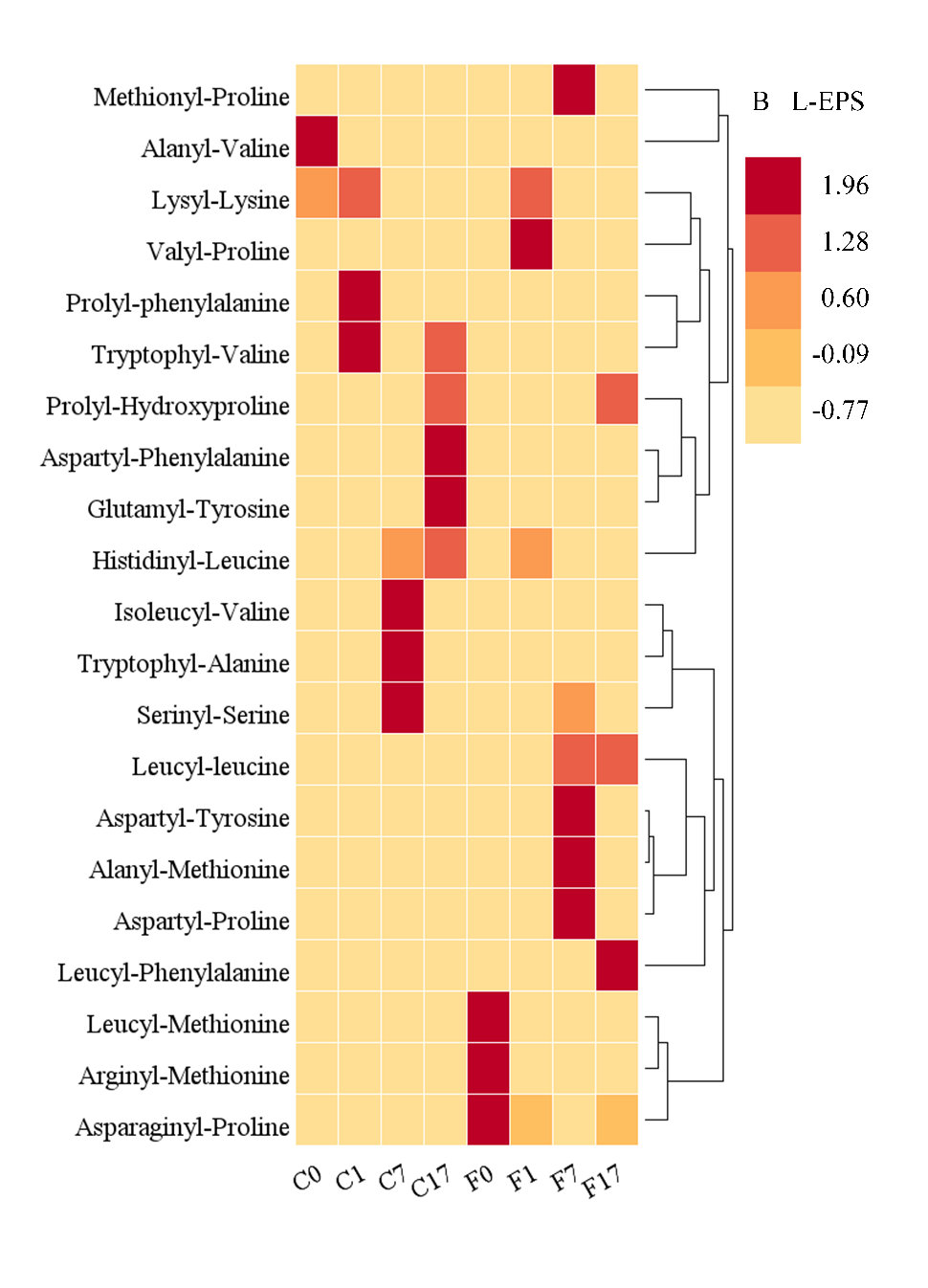


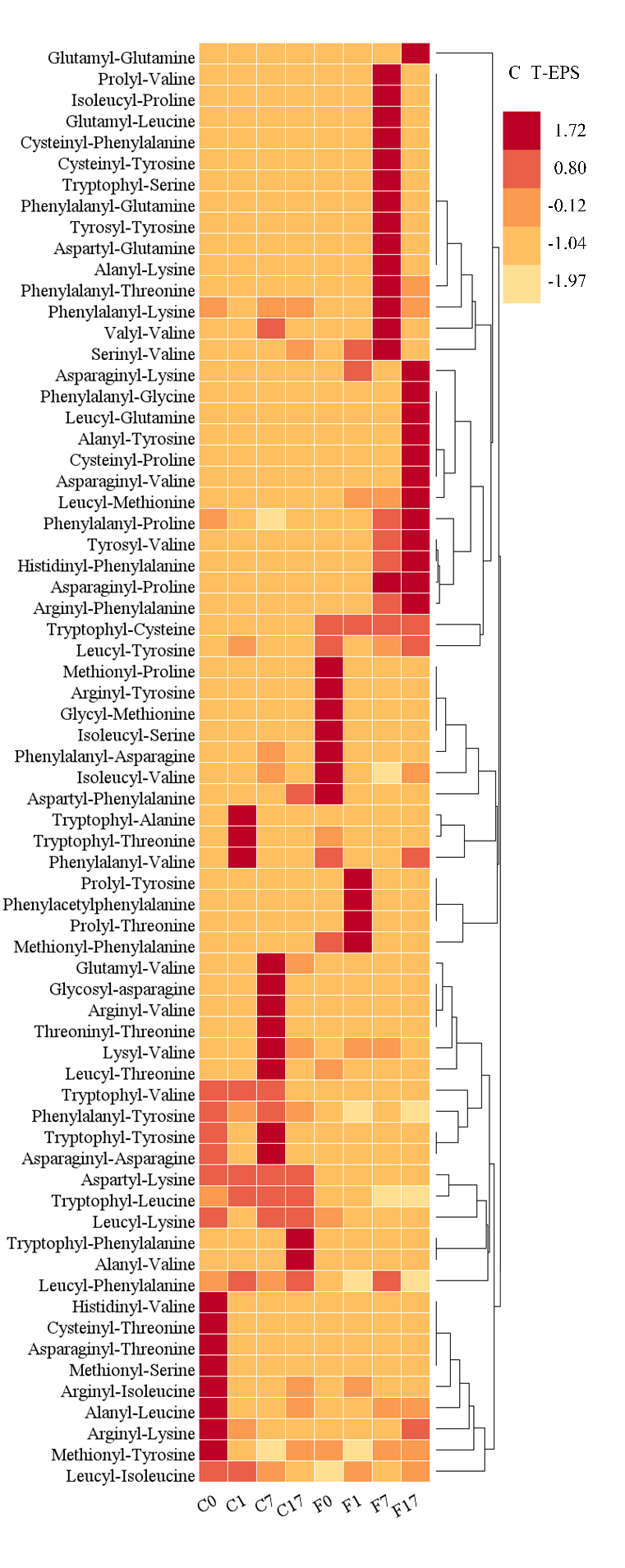


**Figure S4**. Distribution of dipeptides in (A) SMP, (B) L-EPS and (C) T-EPS at different steps in the control and Fe_3_O_4_ reactors.


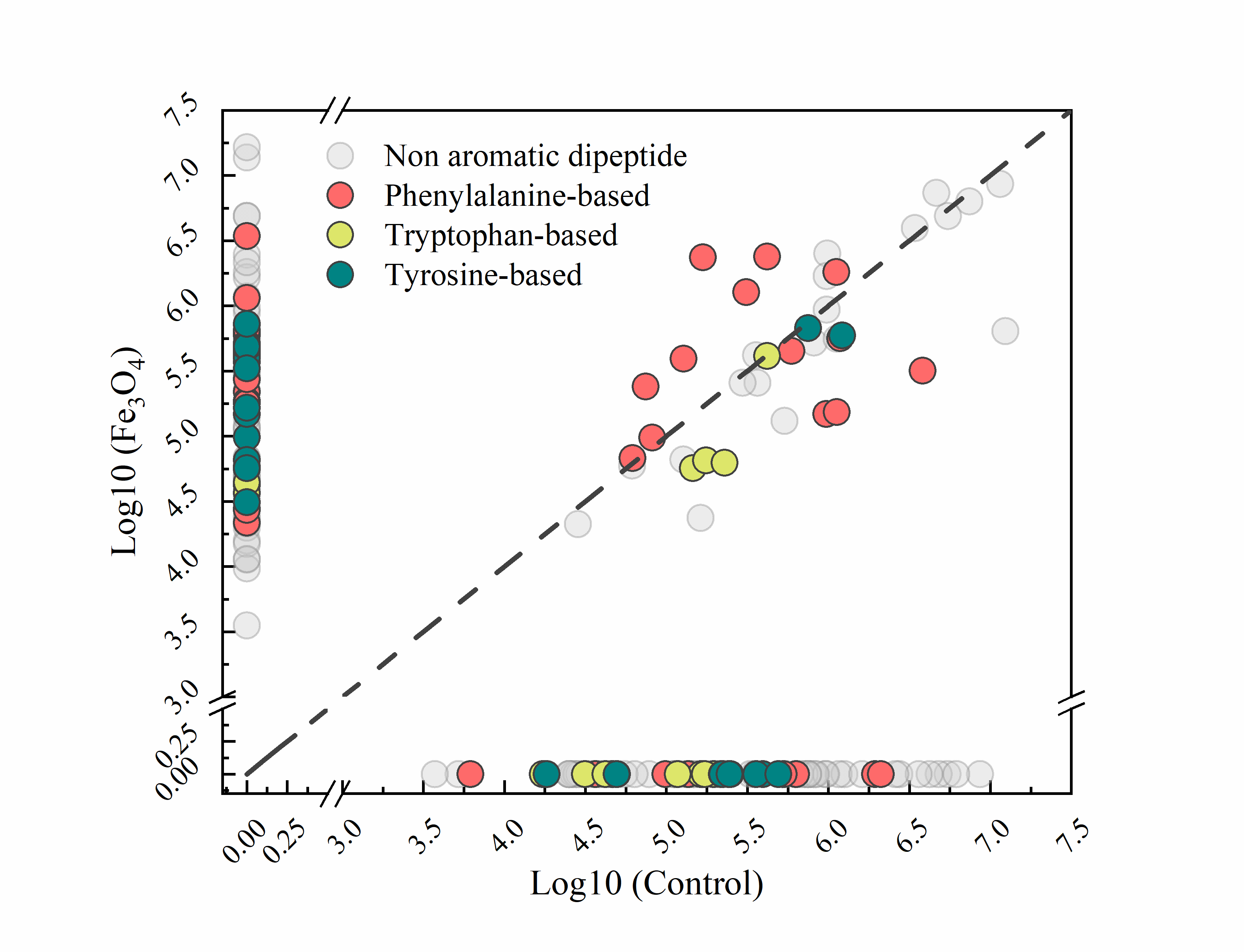


**Figure S5**. Distributed variation of aromatic dipeptides at different steps in the control and Fe_3_O_4_ reactors.


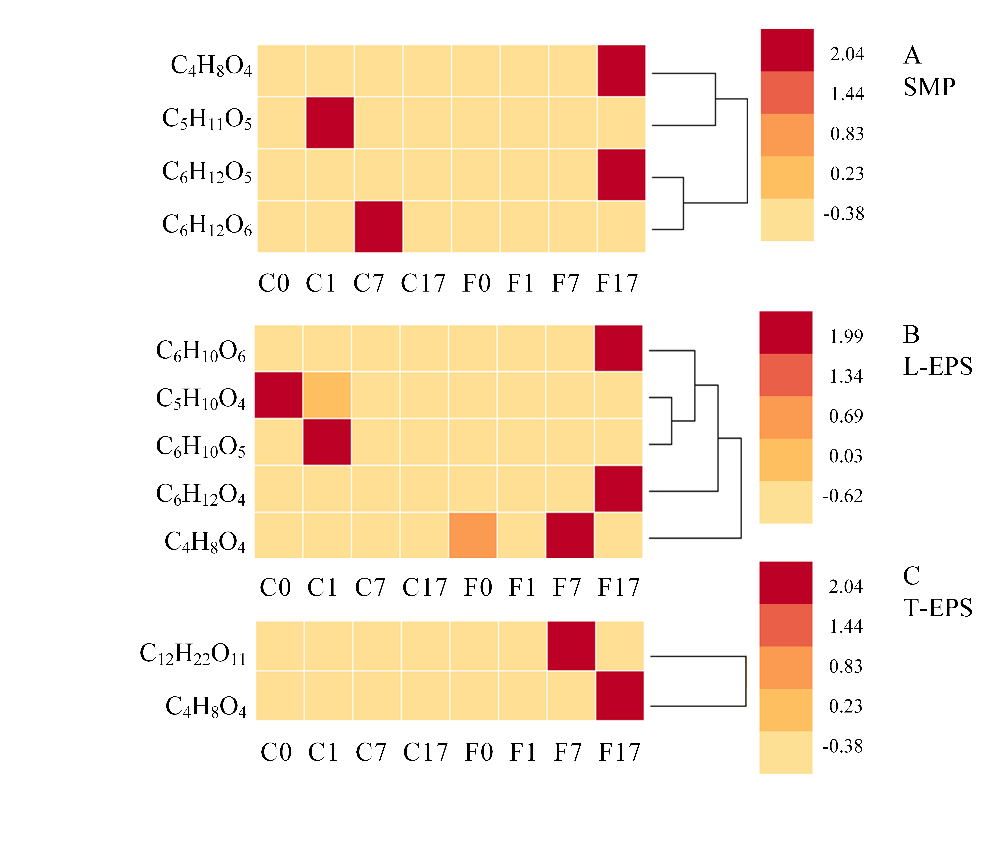


**Figure S6**. Distribution of monosaccharide, disaccharide and derivatives in (A) SMP, (B) L-EPS and (C) T-EPS at different steps in the control and Fe_3_O_4_ reactors.
